# Long-Term Determinants of the Seroprevalence of *Toxoplasma gondii* in a Wild Ungulate Community

**DOI:** 10.3390/ani10122349

**Published:** 2020-12-09

**Authors:** Patricia Barroso, Ignacio García-Bocanegra, Pelayo Acevedo, Pablo Palencia, Francisco Carro, Saúl Jiménez-Ruiz, Sonia Almería, Jitender P. Dubey, David Cano-Terriza, Joaquín Vicente

**Affiliations:** 1 Grupo Sanidad y Biotecnología (SaBio), Instituto de Investigación en Recursos Cinegéticos (IREC) CSIC-UCLM-JCCM, 13071 Ciudad Real, Spain; pelayo.acevedo@uclm.es (P.A.); pablo.palencia@uclm.es (P.P.); saul.jimenez.ruiz@gmail.com (S.J.-R.); joaquin.vicente@uclm.es (J.V.); 2 Grupo de Investigación en Sanidad Animal y Zoonosis (GISAZ), Departamento de Sanidad Animal, Universidad de Córdoba, 14014 Córdoba, Spain; v62garbo@uco.es (I.G.-B.); davidcanovet@gmail.com (D.C.-T.); 3 Estación Biológica Doñana, CSIC, 41092 Sevilla, Spain; pcarro@ebd.csic.es; 4 Division of Virulence Assessment, Office of Applied Research and Safety Assessment (OARSA), Center for Food Safety and Nutrition (CFSAN), Department of Health and Human Services, Food and Drug Administration, Laurel, MD 20708, USA; Maria.Almeria@fda.hhs.gov; 5 Animal Parasitic Disease Laboratory, Beltsville Agricultural Research Center, Agricultural Research Service, United States Department of Agriculture, Building 1001, BARC-East, Beltsville, MD 20705-2350, USA; jitender.dubey@usda.gov; 6 Escuela Técnica Superior de Ingenieros Agrónomos, UCLM, 13071 Ciudad Real, Spain

**Keywords:** parasite, long-term study, protozoan, shared infections, zoonoses, wildlife-livestock interface

## Abstract

**Simple Summary:**

*Toxoplasma gondii* is a zoonotic intracellular parasite which infects a wide range of warm-blooded animals. Long-term studies provide the necessary perspective required to understand those processes which took place over many years in order to address epidemiology and ecology in complex host communities. This study is focused on evaluating what the main long-term determinants of the seroprevalence of *T. gondii* are in the wild ungulate community from Doñana National Park (southwestern Spain). With this purpose, we assayed sera from 1573 wild ungulates (wild boar, red deer, and fallow deer), collected for 13 years (from 2005 to 2018). We found high seroprevalence values of *T. gondii* (% ± CI 95%; wild boar 39 ± 3.3; red deer 30.7 ± 4.4; and fallow deer 29.7 ± 4.2. Several factors operating in the medium and long-term (individual, environmental, population and stochastic) explained the risk of *T. gondii* in wild boar and deer, some of them operating at the community level.

**Abstract:**

*Toxoplasma gondii* is an obligate intracellular protozoan which infects warm-blooded vertebrates, including humans, worldwide. In the present study, the epidemiology of *T. gondii* was studied in the wild ungulate host community (wild boar, red deer, and fallow deer) of Doñana National Park (DNP, south-western Spain) for 13 years (2005–2018). We assessed several variables which potentially operate in the medium and long-term (environmental features, population, and stochastic factors). Overall, the wild ungulate host community of DNP had high seroprevalence values of *T. gondii* (STG; % ± confidence interval (CI) 95%; wild boar (*Sus scrofa*) 39 ± 3.3, *n* = 698; red deer (*Cervus elaphus*) 30.7 ± 4.4, *n* = 423; fallow deer (*Dama dama*) 29.7 ± 4.2, *n* = 452). The complex interplay of hosts and ecological/epidemiological niches, together with the optimal climatic conditions for the survival of oocysts that converge in this area may favor the spread of the parasite in its host community. The temporal evolution of STG oscillated considerably, mostly in deer species. The relationships shown by statistical models indicated that several factors determined species patterns. Concomitance of effects among species, indicated that relevant drivers of risk operated at the community level. Our focus, addressing factors operating at broad temporal scale, allows showing their impacts on the epidemiology of *T. gondii* and its trends. This approach is key to understanding the epidemiology and ecology to *T. gondii* infection in wild host communities in a context where the decline in seroprevalence leads to loss of immunity in humans.

## 1. Introduction

*Toxoplasma gondii* is a zoonotic obligate intracellular protozoan which infects warm-blooded vertebrates [1]. It has an indirect life cycle where wild and domestic felids are the definitive hosts, excreting oocyst in feces. Humans, as well as many mammal and bird species, serve as intermediate hosts of *T. gondii* and can become infected by vertical transmission, the fecal-oral route, through the ingestion of water or food contaminated with sporulated *T. gondii* oocysts, or through the consumption of tissues from animals infected with encysted bradyzoites [1,2].

*T. gondii* has been detected in wildlife and livestock worldwide [1]. Previous Spanish studies revealed a widespread distribution of this parasite in both wild and domestic ungulates, showing significant differences in the presence of *T. gondii* among geographic areas [3,4,5,6,7]. In Mediterranean ecosystems in southern Spain, antibodies against *T. gondii* have been detected in wild ungulates including wild boar (*Sus scrofa*), red deer (*Cervus elaphus*), fallow deer (*Dama dama*), roe deer (*Capreolus capreolus*), Barbary sheep (*Ammotragus lervia*), mouflon (*Ovis aries musimon*) and Iberian ibex (*Capra pyrenaica*). In these studies, seroprevalences of 40.2%, 15.6% and 10.5% were reached in wild boar, fallow deer and red deer, respectively [3,8], being lower or calculated from a few samples in the other species. Regarding livestock, serosurveys in this region revealed rate levels ranging between 18.6–83.3% and 16.2–24.3% in domestic ungulates [5,8,9] and pigs, respectively [7,10,11]. Higher seroprevalences from this area have been reported in wild carnivores, especially in the Iberian lynx (*Lynx pardinus*), reaching rates of 81.5% [12].

Host-pathogen dynamics are subjected to several processes which operate over broad temporal scales; however, little attention has been paid to *T. gondii*, and particularly, in intermediate host communities at the wildlife-livestock interface [4,8]. Wide temporal data series are essential to address epidemiology and ecology in complex host communities with the necessary perspective required to understand processes taking place over many years [13,14,15,16]. In Doñana National Park (DNP, South West Spain), the wild ungulate community (including wild boar, red deer and fallow deer) occurs sympatrically with free-ranging cattle and horses, and one of the most important meta-population of the endangered Iberian lynx [17]. Studies on *T. gondii* in DNP has been exclusively conducted in felid populations with conservational purposes, showing a widespread infection in the area and reporting seroprevalence rates up to 60% [12,18,19].

The multiple transmission routes and capacity of *T. gondii* to find niches into the hosts studied provided an excellent scenario to improve our understanding of the transmission dynamics of this pathogen. While *T. gondii* has normally been considered an excellent model to study host-pathogen interactions, we also showed that it may also be used to address the study of population, community and environmental factors. The present long-term study illustrates the interplay of factors, particularly factors operating at broad temporal scale that may contribute to the spread and maintenance of a pathogen over host communities. In this context, we present data on serosurveillance of *T. gondii* in wild ungulates (wild boar, red deer, and fallow deer) from DNP for a 13-years, with the specific aims of: (I) evaluating the factors (individual, populational and environmental) modulating the seroprevalence of *T. gondii* (STG), and (II) assessing the factors operating in the long-term (population and stochastic) in order to explain the temporal trend of STG in the intermediate host ungulate community from 2005 to 2018.

## 2. Materials and Methods

### 2.1. Study Area

This study was conducted in DNP (54,000 ha), one of the most relevant biodiversity reserves in Europe, located on the Atlantic coast of southwestern Spain (37°09′ N, 6°30′ W). Human access to the park is restricted and agriculture and hunting are prohibited inside the park; cattle and horse breeding are allowed and are, mainly focused on autochthonous and traditional breeds [20].

**Figure 1:**
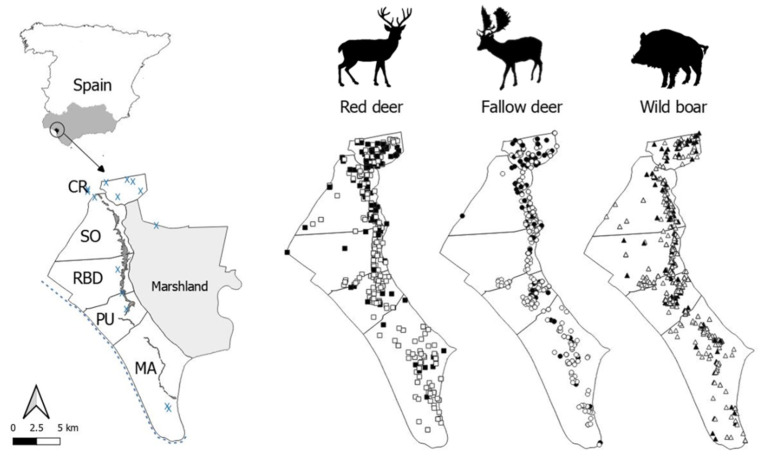
Map of the study area, Doñana National Park. The sampling areas (cattle management units: Coto del Rey (CR), Sotos (SO), Doñana Biological Reserve (RBD), Puntal (PU) and Marismillas (MA)) are delimited and the ecotone and small human settlements are displayed by a dark band and blue “X”, respectively. Red deer (squares), fallow deer (circles), and wild boar (triangles) sampled are shown. Black and white symbols mean animals positive and negative for antibodies against *Toxoplasma gondii*, respectively.

The habitat consists of a greater proportion of sand dune habitat and marshland, combined with pine forest and Mediterranean scrubland (see Figure 1 and [21] for a more detailed description). Between the scrublands and the edge of the marshland, there is a narrow north-south longitudinal strip of humid ecotone of high ecological richness.

DNP has a dry sub-humid Mediterranean climate with strong seasonality, especially in terms of water availability to animals and vegetation. The average annual temperature is 17 °C, and the mean annual precipitation is 550 mm, with high intra and interannual fluctuation (170 to 1000 mm), which determine the dynamics of the marshlands [22]. During the wet seasons (winter and spring) the marshlands may flood, so ungulates concentrate and browse in the remaining uncovered scrublands. In late summer and autumn, the hardest season for ungulates due to the shortage of resources and the seasonal drought, an aggregation of wild and domestic ungulates on the ecotone and around water points occurs [21].

The territory of DNP included in this study is divided into five cattle management areas from north to south: Coto del Rey (CR), Sotos (SO), Doñana Biological Reserve (RBD), Puntal (PU) and Marismillas (MA). Free-ranging livestock is distributed through the entire park, except in the northernmost area (CR). In this area, despite the existence of a low number of horses since the last year, cattle husbandry is prohibited since 2002 as a conservation measure for the endangered Iberian lynx. A meta-population of 94 individuals of Iberian lynx currently inhabit DNP and the surrounding areas [17]. The remaining community of carnivores is comprised by red fox (*Vulpes vulpes*), Eurasian badger (*Meles meles*), Eurasian otter (*Lutra lutra*), polecat (*Mustela putorius*), European genet (*Genetta genetta*), Egyptian mongoose (*Herpestes ichneumon*), and occasionally, wild cat (*Felis silvestris silvestris*), whose presence is very scarce, probably due to the presence of a larger predator such as the Iberian lynx [23]. Furthermore, domestic carnivores including stray cats (*Felis silvestris catus*) and dogs (*Canis lupus familiaris*) are also occasionally present throughout DNP, although a population control plan of stray dogs and cats has been carried out in DNP since 2007 [24].

Finally, population control (by culling) of the wild ungulate population is practiced exclusively by park rangers as part of the park management scheme, and it is also used to carry out a health-monitoring program [14].

### 2.2. Animal Sampling

From October–January of 2005 to 2018 (sampling seasons 2005–2006 to 2017–2018), 423 red deer, 452 fallow deer and 698 wild boar were randomly (in terms of sex, age and health status) sampled in the population control context performed by park rangers and necropsied as part of the DNP health-monitoring program (approved by the Research Commission of DNP in accordance with management rules established by the Autonomous Government of Andalusia). Table S1 displays the sample size by species, sampling site and season, as well as the seroprevalences found. For each individual, the geographical location of the sighting was recorded through a portable GPS (Garmin Ltd., Olathe, KS, USA).

The sampling was performed according to European (EC Directive 86/609/EEC; [25]) and Spanish laws (RD 223/1988; [26]), current guidelines for the ethical use of animals in research [27], the Animal Experiment Committee of Castilla-La Mancha University and the Spanish Ethics Committee (PR-2015-03-08). Necropsies and sample collection were undertaken in the field by qualified veterinarians. During the examination, blood samples were collected into sterile plastic tubes (Vacutainer^®^, Becton-Dickinson, NJ, USA) from the heart, thoracic cavity, or preferably by endocranial venous sinuses puncture [28].

### 2.3. Serological Testing

Sera were obtained after centrifugation at 40× *g* for 5 min and stored at −20 °C until assayed for antibodies. Antibodies to *T. gondii* were tested using the modified agglutination test (MAT) as previously described [29]. This technique has been employed broadly for the diagnosis of antibodies against *T. gondii* in both domestic and wildlife species [1]. Two recent large studies in wild pigs and white-tailed deer in the USA added evidence for the validity of serological analysis by MAT in those species since viable *T. gondii* was isolated from a large number of seropositive animals and the rate of isolating viable parasites was positively associated with MAT titers in those studies [30,31,32,33]. Each serum sample was tested at 1:25 and 1:50 dilutions, including positive and negative controls in each test. Sera with a titer of 1:25 or higher were considered positive and those with doubtful or positive results were re-tested [12,34,35].

### 2.4. Data Collection

#### 2.4.1. Individual Factors

The sex and age of the animals were determined, classifying them into three age classes on the basis of dentition eruption patterns [36]: calves (<1-year-old), juveniles (1–2 years) and adults (≥3 years) for deer species, and piglets (<6 months), juveniles (0.5–2 years) and adults (>2 years) for wild boar.

Considering the well-known debilitating effect of tuberculosis (TB) progression on immune response [37], we assessed the potential effect of TB severity on the seroprevalence against *T. gondii*. For this purpose, the presence of concomitant tuberculosis-like lesions (TBL) was used as a proxy to infection by the *Mycobacterium tuberculosis* Complex (MTC), since it provides a relatively accurate diagnosis without the need for expensive laboratory confirmation [14,15]. The presence of TBL was recorded by macroscopic inspection of the head, thoracic and mesenteric lymph nodes as well as abdominal and thoracic organs in the laboratory (see [38] for a detailed methodology). When TBL are identified in at least two of the three anatomical compartments examined (head, thorax, and abdomen) we considered the TBL as generalized, indicative of a more severe and evolved infection [38]. According to the generalized TBL status, wild ungulates were grouped in two classes: those without TBL or showing localized TBL in a single anatomical compartment, and a second class including animals with presence of generalized TBL.

#### 2.4.2. Environmental Factors

As for environmental information, several variables were included in our analysis to assess their effect on STG because of their importance to ungulate behavior, distribution, and transmission of pathogens in DNP and South Spain [15,21,39]. A grid of one hectare of surface was built, generating territorial units in which the proportional cover of dense scrub, low-clear shrubland, herbaceous grassland, woodland, bare land and watercourse vegetation were calculated for each territorial unit (see [40]). This grid was merged with the geographical location of the animals through a point sampling tool with QGIS version 2.12.1 [41]. Landcover data was obtained from Andalusia Environmental Information [42].

Given the effects reportedly associated with urban areas [43], the coast [44] and surface water on the infection risk of *T. gondii* [45], the effect of the nearest location of animals to these areas was assessed. For that purpose, we calculated the straight-line distance (m) from the exact location of each animal sampled to the nearest: urban area (DURB), small human settlements (DHS), coast line (DCOAST), water point (DWAT) and marsh-shrub ecotone (DE) (see [14,21,46]).

#### 2.4.3. Population Factors

To estimate the population density of wild ungulates we applied distance sampling methodology [47]. Every year during September, and two hours before sunset, we sampled twice 7 line transects of 10–15 km each one, distributed throughout the study area. Additionally, for wild boar we repeated the transect one hour after sunset in order to increase the sample size. We carried out the surveys during September because it is the month of maximum detectability for these species [48], and to obtain density results just before the health-monitoring program. Moreover, during September the marshland was dry, and it allowed us to sample all the habitats in DNP. The surveys were carried out from a vehicle (average speed was 10 km/h), and the perpendicular distance between animals and transect was recorded with a telemeter (Garmin Ltd., Olathe, KS, USA). The analysis were carried out using Distance Sampling 6.2 software [49] by considering stratification. We defined three strata according to its abundance and visibility: shrubland, marshland and ecotone. The data of all the years (2005–2018) were considered to estimate a detection function for each stratum, and we considered the data of each strata, sampling season and livestock management area to estimate the encounter rate and mean group size. Data were right-truncated when the probability of detection was lower than 0.15 [47]. Half-normal, uniform and hazard rate models for the detection function were fitted against the data using cosine, hermite polynomial, and simple polynomial adjustment terms, fitted sequentially. The selection of the best model was based on the Akaike’s Information Criterion (AIC) [50].

The abundance of the diverse community of carnivores from DNP was monitored by means of track surveys along prefixed transects on sandy substrate according monitoring team program (ESPN-EBD-CSIC). Tracks left on moist sand over a 24 h period were tracked in transects of 1.5 m width and 2 km length, from dawn to midday and were expressed as Kilometric Abundance Index (KAI) of footprints. The surveys include 12 different transects distributed across the DNP which were repeated during three consecutive days, being cleaned daily.

As for livestock, we calculated the cattle and horse stocks per square kilometer for each sampling site and season.

#### 2.4.4. Stochastic Factors

Meteorological information (average rainfall and temperature) was collected from the meteorology station located at RBD for each sampling season [51]. In Mediterranean environments, rainfall and temperature have potential relevance to the dynamics of ungulate populations, as well as effects on the susceptibility or exposure to pathogens [15]. Specifically for *T. gondii*, both factors are key for the survival of oocyst in the environment [52]. Therefore, they were considered here for their potential effect in *T. gondii* epidemiology.

### 2.5. Risk Factor Analysis

Initially, collinearity between environmental and population variables was explored [53]. Given the high collinearity observed between environmental variables and with the purpose of simplifying the environmental information, a principal component analysis (PCA) was performed, obtaining two uncorrelated environmental factors: closed habitats, in which dense scrub and woodlands predominates, and watercourse habitats in which watercourse vegetation predominates.

Generalized linear mixed models (GzLMMs; binomial family) were used to assess the effect of the range of explanatory variables on the individual serological status against *T. gondii* (negative/positive). The statistical differences in STG among sampling areas (CR, SO, RBD, PU and MA) were evaluated in a first exploratory approach, the purpose of which was showing spatial differences in the serological status against *T. gondii*. A GzLMM for each species (red deer, fallow deer, and wild boar) was designed. In these models, serological status against *T. gondii* was the response variable; the sex, age class, and the sampling area were the explanatory variables. The sampling season and month were fitted in the model as random-effect factors.

Concerning the final model, it included sampling area and season as random-effect factors, since the main aim of this study was to generalize the effect of the variables included on the serological status against *T. gondii* regardless of the sampling area. Models were also performed separately for each species (red deer, fallow deer, and wild boar). The explanatory variables included individual, environmental, population and stochastic factors. Individual factors encompassed sex, age class, and tuberculosis status. Environmental factors comprised DWAT, DE, DCOAST, DHS, DURB, closed habitats, and watercourse habitats. Regarding populational factors, the population densities of wild (fallow deer, red deer, and wild boar) and domestic ungulates (cattle and horses), as well as the abundance (KAI of footprints) of wild carnivores (all together genet, Eurasian badger, red fox, and Egyptian mongoose, and separately, the abundance of Iberian lynx) were included. Finally, the stochastic factors were the previous seasons’ rainfall and temperature. The two-way interactions between individual-stochastic factors separately (sex-age, and previous season’s rainfall-temperature) and all together (sex-rainfall and age-rainfall), as well as between population-individual factors (density-age), and population-stochastic factors (previous season´s rainfall-density) were also included in the models. For the GzLMMs, a binomial error and a logit link function were used. Stepwise selection processes for the final models were performed on the basis of the AIC [50] (Table S2). Furthermore, the assumptions of binomial GzLMMs were met in all the best models selected [53]. The predicted probabilities of serological response to *T. gondii* obtained from these models were used to represent the results. Finally, cross-correlations and autocorrelations between STG and its predicted response probability between the different species were carried out to explore similarities of temporal patterns [54].

The statistical analyses were performed using R-studio software version 4.0.2 [55]. All models were performed using the R package glmer [56]. Significant *p*-values were set at 0.05.

## 3. Results

### 3.1. General

**Figure 2:**
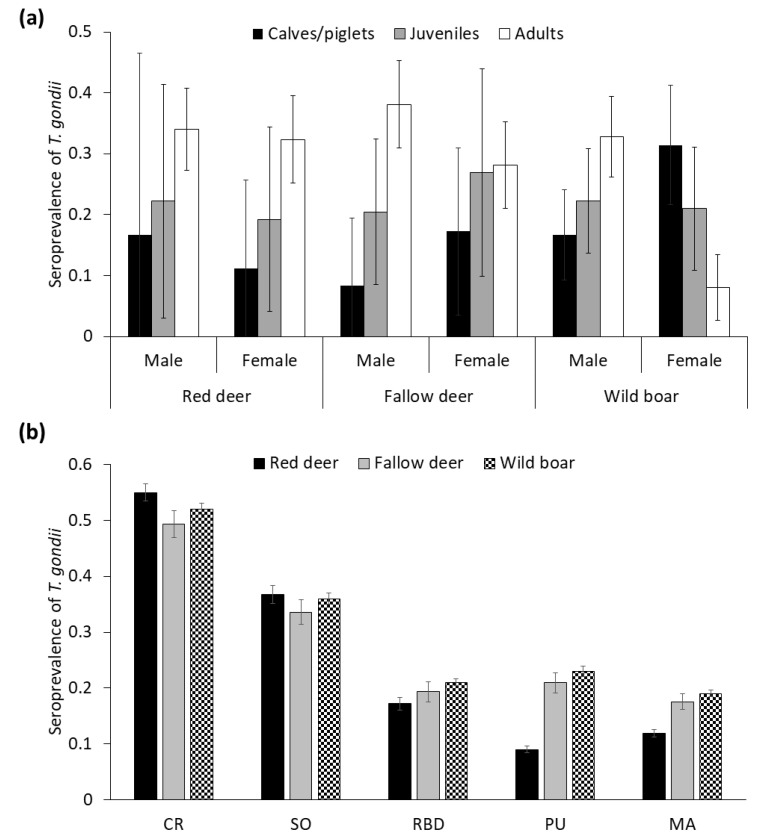
(**a**) Seroprevalence (±CI 95%) of *Toxoplasma gondii* depending on age class and sex in red deer, fallow deer and wild boar (**b**) Seroprevalence (±CI 95%) of *T. gondii* obtained from selected generalized linear mixed models (GzLMMs) for the species studied depending on the sampling area, from north to south areas (see Figure 1 for a map of the areas with their full names).

The STG (MAT ≥ 1:25; % ± confidence interval (CI) 95%) in wild boar was 39.0 ± 3.3 (*n* = 698), followed by red deer 30.7 ± 4.4 (*n* = 423), and fallow deer 29.7 ± 4.2 (*n* = 452). Among the seropositive animals, titers of 1:25 were detected in 34.5% wild boar, 27.7% red deer, and 55.2% fallow deer, whereas titers ≥ 1:50 were found in 72.3% red deer, 65.5% wild boar, and 44.8% fallow deer. We observed increasing age trends in STG in all wild ungulate species, except for wild boar females (Figure 2a) since, interestingly, piglets already showed high STG. With respect to gender, males tended to present higher STG than females in deer species (32.1–28.8% and 32–26.4% for red deer and fallow deer, respectively), whereas the opposite was observed in wild boar (STG = 26.2% for males, STG = 29.7% for females; Figure 2a; see statistical comparisons below).

**Figure 3:**
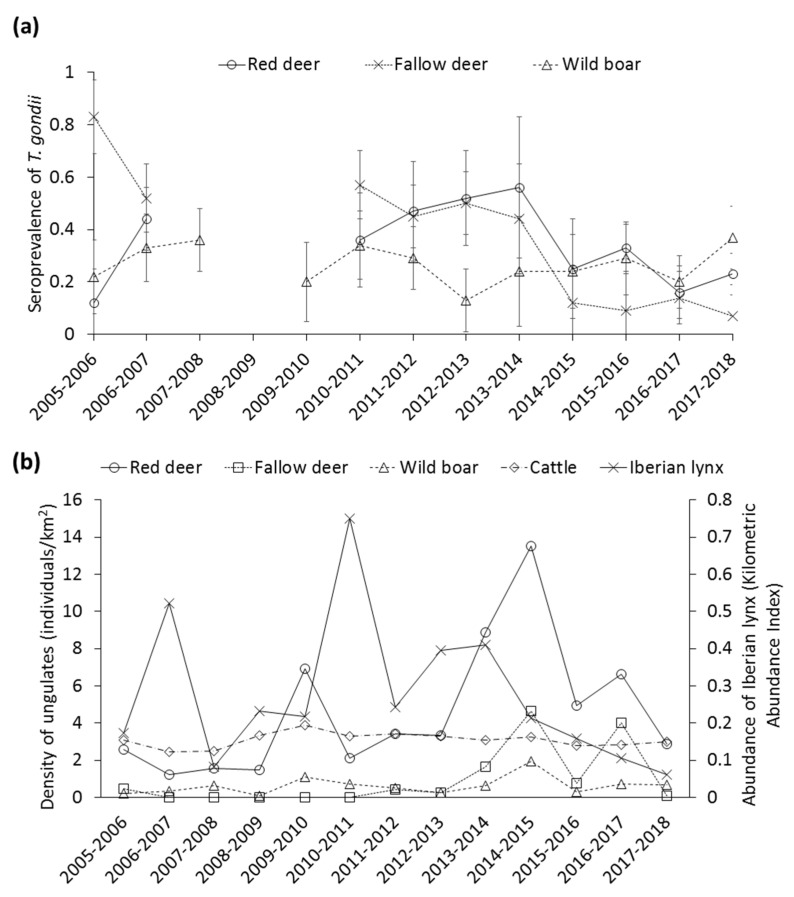
Temporal trend of the (**a**) seroprevalence of *Toxoplasma gondii* (±CI 95%), and (**b**) population density of red deer, fallow deer, wild boar, and cattle (individuals/km^2^), and Kilometric Abundance Index of Iberian lynx.

Contrasted STG were apparent among areas, which was consistent across species. In this sense, seroprevalence decreased from north to south, more markedly in red deer (Figure 2b). The temporal evolution of STG, and trends in the estimated density/abundance of each different species are summarized in Figure 3a,b, respectively. In this regard, the STG exhibited strong annual fluctuations, mostly in deer species (Figure 3a). Actually, it is noteworthy the significant decrease of STG in these species since the season 2013–2014. No autocorrelations or cross-correlations were observed.

### 3.2. Factors Determining the Seroprevalence of T. gondii

There were statistically significant differences in the STG between sampling areas for all the wild ungulates species (red deer, *F* = 13.4, df = 410, *p* ≤ 0.01; fallow deer, *F* = 4.5, df = 436, *p* ≤ 0.01; and wild boar *F* = 10.3, df = 682, *p* ≤ 0.01), confirming the north to south spatial decreasing gradient (Figure 2b).

The results of the GzLMMs on the status against *T. gondii*, incorporating broader environmental and populational information are shown in Table 1. The conditional R^2^ obtained from these models were 0.37, 0.53 and 0.25, for red deer, fallow deer, and wild boar, respectively.

#### 3.2.1. Individual Factors

The sex and age classes were statistically significant factors in the models on red deer and wild boar. However, no sex or age-related differences were found in fallow deer. Regarding red deer, females had lower STG than males, and irrespective of sex, the pattern increased with the age. Concerning wild boar, different sex-related age patterns were shown, increasing for males but not for females (the sex by age interaction was marginally significant).

**Figure 4:**
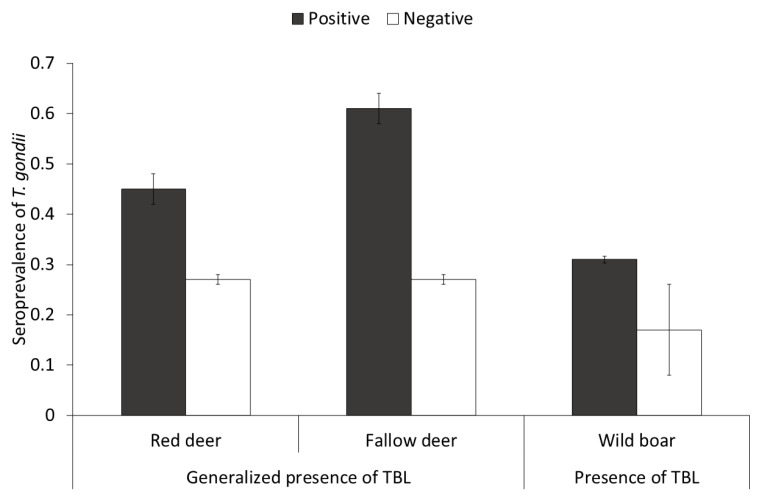
Seroprevalence (±CI 95%) of *Toxoplasma gondii* depending on the tuberculosis status in red deer and fallow deer (interpreted as positive animals with generalized presence of tuberculosis-like lesions (TBL), and in wild boar (interpreted as positive animals with presence of TBL).

Regarding TB status, the prevalence of TBL (% ± CI 95%) for wild boar, red deer and fallow deer were 77.4 ± 3.1, 42.5 ± 4.7, and 16.4 ± 3.7, respectively. Wild boar had the highest prevalence of generalized TBL (% ± CI 95%; 27.73 ± 3.5), followed by red deer (17.7 ± 4.2) and fallow deer (8.11 ± 2.9). The STG was higher in red deer and fallow deer presenting generalized TBL (Figure 4) compared to generalized TBL-free individuals (TBL-free plus not generalized TBL positive). As for the wild boar, a complementary model was performed with the purpose of exploring the effect of the presence of TBL (positive or negative), since no effect of the presence of generalized TBL was observed. In this model, wild boar presenting TBL showed higher STG than negative individuals (*F* = 8.96, df = 695, *p* = 0.05).

#### 3.2.2. Environmental Factors

**Figure 5:**
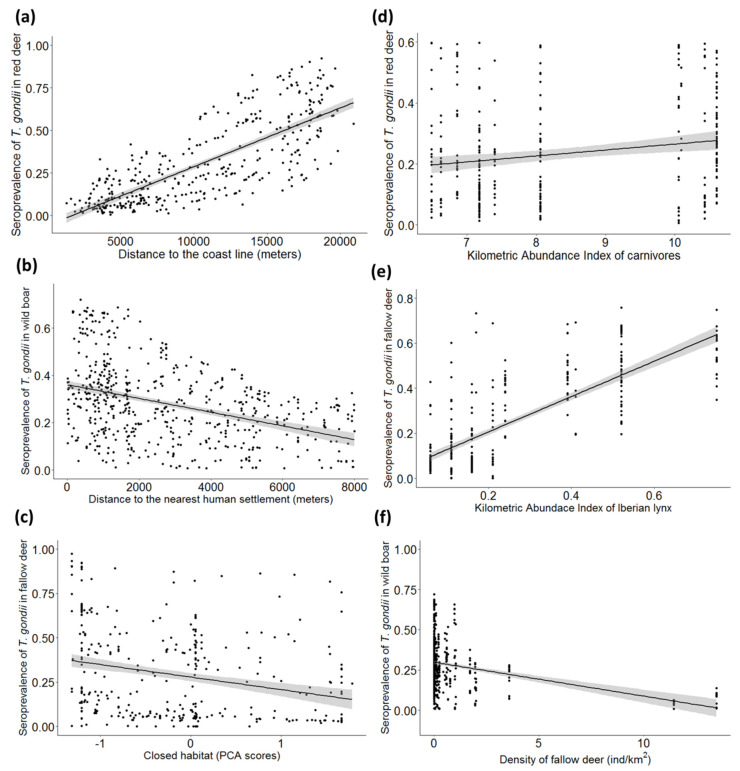
Seroprevalence (±CI 95%, represented by the shaded band) of *Toxoplasma gondii* obtained from selected generalized linear mixed models (GzLMMs) in (**a**) red deer depending on the distance to the coast line (m), (**b**) wild boar depending on the distance to the nearest human settlement (m), (**c**) fallow deer depending on the cover level of closed habitats, measured according to the principal component analysis (PCA) scores from axis 1, (**d**) red deer depending on the Kilometric Abundance Index of carnivores species (KAI), (**e**) fallow deer depending on the Kilometric Abundance Index of Iberian lynx (KAI), and (**f**) wild boar depending on the density of fallow deer (individuals/km^2^).

The further to the coastline, the higher the STG was (see e.g., Figure 5a for red deer) in all the species. Moreover, the closer to small human settlements, the higher the STG for wild boar was (Figure 5b). The increased availability of closed habitat significantly associated with lower STG in fallow deer (Figure 5c).

#### 3.2.3. Population Factors

The abundance of carnivores significantly and positively associated with the exposure to *T. gondii* in red deer (Figure 5d), and similarly, the Iberian lynx abundance positively associated with the seropositivity to this parasite in fallow deer (Figure 5e). The fallow deer density negatively associated with the STG in wild boar (Figure 5f) and fallow deer, but positively in the case of red deer. As for red deer, a negative association was found between STG and density. In contrast, wild boar showed higher STG at higher densities.

#### 3.2.4. Stochastic Factors

**Figure 6:**
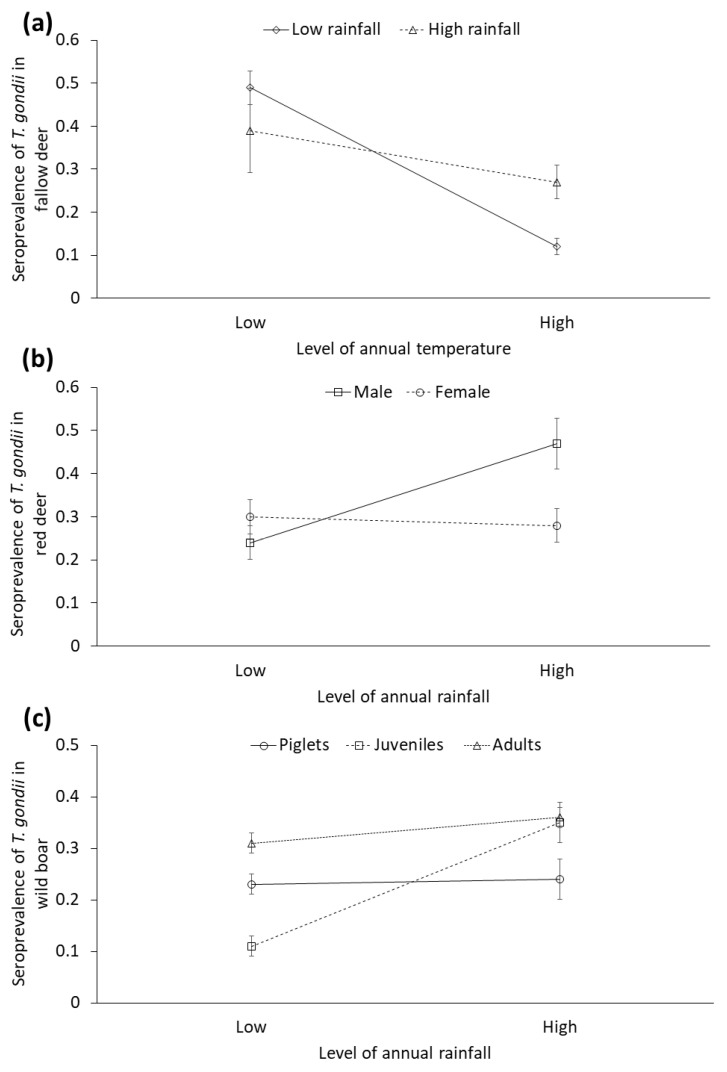
Seroprevalence (±CI 95%) of *Toxoplasma gondii* obtained from selected generalized linear mixed models (GzLMMs) in (**a**) fallow deer depending on the interaction between annual rainfall (mm) and temperature (°C), (**b**) red deer depending on the interaction between annual rainfall (mm) and sex, and (**c**) wild boar depending on the interaction between annual rainfall (mm) and age.

To represent our results, and considering the mean values obtained, we established the following categories of rainfall and temperature for displaying results: low rainfall (≤521.10 mm), high rainfall (>521.10 mm), low temperature (≤17.5 °C) and high temperature (>17.5 °C). Lower annual temperature was associated with higher STG in red deer. Furthermore, higher annual rainfall was associated with higher seropositivity to *T. gondii* in red deer. Regarding fallow deer, the interaction between rainfall and temperature was significant: overall, there was a trend to higher STG in cold years, and this pattern was more marked in dry years (see Figure 6a). Rainy years were statistically associated with higher STG in male red deer, but not in females (significant rainfall by sex interaction, Figure 6b). Concerning the wild boar, the rainfall was positively associated with the STG in juveniles, but this effect was not shown in other age classes (significant annual rainfall by age interaction, Figure 6c).

## 4. Discussion

### 4.1. General Patterns of the Seroprevalence of T. gondii

The STG reported in the present study oscillated considerably (from 29.7 to 39%) between the three species tested and sharing the same environment. This may be caused by differences in the susceptibility, the feeding behavior, or the habitat use of those species determining the exposure [4,52]. The seroprevalence detected in wild boar (39%) concurs with studies conducted in Europe [3,57,58,59]. However, most studies from European countries, STG in wild boar ranged from 6 to 25% [6,60,61]. In this regard, trophic relationships by predation and/or scavenging of a wide range of warm-blooded animals of the DNP may operate.

Concerning deer, the STG obtained (30.7% and 29.7%, for red deer and fallow deer, respectively) are also in accordance with those reported in the literature over Europe in general [62,63] and Spain in particular [8,34], ranging from 10.5 to 48%. Specifically for fallow deer, STG (29.7%) were among the highest reported in European studies that were mainly focused on Spain [8,34,63]. It was only exceeded by the rates obtained by Calero-Bernal et al. [64] in south-central Spain (reaching the 48%). The higher rate of movement between areas reported for fallow deer in DNP may imply higher exposure to *T. gondii*, explaining the high STG observed in this species [39].

Overall, the wild ungulate host community of DNP showed higher STG compared with those reported in the literature of the European and Iberian contexts [34,61,62]. Mechanisms determining seroprevalence in different host species of the studied community are related to the life cycle of *T. gondii*, which involves both an environmental and a trophic transmission route (i.e., trophic relationships among potential hosts of the community; [65]). Terrestrial herbivores should have the lowest *T. gondii* exposure, only through the ingestion of oocyst-contaminated vegetation, soil and/or drinking water. In DNP, the environmental presence of oocyst excreted by felids may be playing a major role (see below). The high biodiversity inhabiting DNP, which provides a wide range of hosts and ecological/epidemiological niches, and the optimal climatic conditions for the survival of the oocysts may favor the spread of the parasite in the DNP host community.

The specific role of the different factors in a long-term perspective is also detailed further in the discussion. Interestingly, while the STG exhibited strong annual fluctuations, mostly in deer species, it was more stable in wild boar. In populations from The Netherlands, seroprevalence in wild boar similarly established at around 35% [66]. Whereas authors stated that the actual mechanisms behind the stabilization requires further investigation, an epidemiological SIS-model that included a reversion to susceptible after infection (with loss of antibodies that may have been preceded by a loss of tissue cysts), fitted the data much better.

The north to south spatial gradient observed is similar to that exhibited by the prevalence of other shared pathogens tested in the wild ungulate community of DNP in previous studies [14,67]. This pattern may relate to spatial variation in the contamination of the environment by *T. gondii* oocyst. The main large human settlements around DNP are concentrated in the northern part of the park, with a subsequent higher presence of peri-domestic cat populations [68], which may contaminate the environment with oocysts. Iberian lynx populations also show a north to south decreasing pattern in DNP [69], contributing to a lesser extent to this contamination. *T. gondii* oocysts were found in feces of 17% of cats sharing a habitat with Iberian lynx [19]. In this regard, feral cats are the more likely reservoir host of parasites affecting the Iberian lynx and wildlife species in general, especially in areas where feral cats are abundant and widespread such as DNP surroundings [70,71].

### 4.2. Individual Factors

In wild boar, overall, females had significantly higher STG than males. This result is in accordance with previous studies in this species [3,57,58,59]. However, the age pattern observed was opposite to that of males. Several authors have reported that no statistically significant effect of age on STG in wild boar was observed [3,72,73], whereas only one study found a significantly higher prevalence in adult wild boar [59]. Nevertheless, we must consider sex by age pattern to understand the differences. The increased exposure to *T. gondii* through life, together with the high persistence of antibodies against *T. gondii*, could explain the age pattern found in the STG in males. Even so, in females, the decline in the STG rather than indicating a decrease of exposure to the parasite, may be indicative of a subtle equilibrium of chronic infection and reduced specific humoral response that is not detected. Ecological and evolutive aspects determining differences in exposure may be behind this pattern. However, further research is required. Finally, piglets exhibiting high seroprevalences could be explained by maternal-derived antibodies, whose titers depend on those shown by sows, according to previous studies [10].

As for the red deer, the STG was significantly higher in adult individuals, which has been previously reported in many studies on *T. gondii* [8,57,58,73]. An apparent similar trend, but not significant, was observed in fallow deer (Figure 2a). The increased exposure to *T. gondii* along life together with the high persistence of antibodies against *T. gondii* could explain the age pattern found in deer species.

Concerning concomitant TB infection, overall, the positive TB status of the animals significantly associated with STG in all the species studied. For deer species, the generalized presence of TBL was relevant, as well as the presence of TBL for wild boar. There are several studies on TB-*T. gondii* co-infection in humans [74,75] but not in animals. The relationship between TB and STG observed may be mediated by exposure over time (age-related) and environment. In the latter case, the conditions favoring the persistence of MTC and *T. gondii* oocyst in the environment are similar (see Section 4.3 and Section 4.5).

### 4.3. Environmental Factors

The distance to the coastline significantly positively associated with STG for all wild ungulates. Despite the usually reported contamination of seawater with *T. gondii* [44], less favorable conditions to the survival of oocysts may occur in the surroundings of the coastline, according to previous studies developed in northern Spain [4]. These conditions are mainly the high temperatures reached in the sandy soils which favor the desiccation of the oocysts. The availability of closed habitat (more covered by vegetation) negatively associated with STG in fallow deer (see Figure 5c). This species typically uses and occupies meadows in the park, and individuals sampled in more densely covered areas may have experienced lower exposure to *T. gondii*. By contrast, individuals of the other ungulate species combine the use of both types of habitats which may determine the absence of this effect.

The closer to small human settlements the higher the STG was for wild boar (see Figure 5b). This result is in accordance with previous studies where human settlements have become areas of epidemiological relevance for *T. gondii* infection, mainly mediated by the presence of peri-domestic species [4,45]. There are several human dwellings inside the park, around which peri-domestic cats could settle and consequently contaminate the surroundings of these areas with oocysts. Moreover, wild boar could become infected through the consumption of food scraps from garbage located in these small settlements.

### 4.4. Population Factors

The abundances of Iberian lynx and other carnivores were statistically positively associated with STG in deer species. The presence and abundance of felids have been considered a relevant risk factor associated with *T. gondii* in livestock and wildlife worldwide [1,7,8,9,10]. It has been reported that Iberian lynx could prey on fallow deer and less frequently on juveniles of red deer during seasons of rabbit´s scarceness in DNP, especially in winter and autumn, reaching 5–10% of the biomass in the Iberian lynx diet [76]. This leads to increased environmental contamination with *T. gondii* in areas with presence of wild ungulates. Furthermore, *T. gondii* infected red deer carcasses could pose a potential risk of *T. gondii* infection for carnivores species via scavenging and may therefore play a role as an amplifier of infection in the community [63]. This allows *T. gondii* to finish its life cycle, perpetuating its maintenance in the DNP host community. Little is known about the abundance of small mammals, as well as their STG in DNP. Further studies should focus on investigating their role in the maintenance and spread of this parasite in DNP.

STG significantly increases with density in wild boar. Several authors have described *T. gondii* as a density-dependent parasite for swine [3,45]. Density, together with ecological and behavioral factors typical of this species, could determine increased exposure by wild boar. Scavenging (including cannibalism) may be increased in high density situations when resources are scarce. In the dry season, both the availability of carcasses and exacerbated cannibalism behavior [77], but also ingestion of rodents and birds occur [78]. The negative relationship found both in red deer and fallow deer between density and the risk to test positive can be explained by high recruitment of susceptible individuals (non-infected offspring) associated with high density years. Moreover, unlike for wild boar, this negative association indicates that no density-dependent effect in *T. gondii* infection occurs in deer species. These species become infected only through the ingestion of water or food contaminated with sporulated *T. gondii* oocysts, so no direct transmission route exists as in the case of wild boar, which possesses scavenging habits. However, the positive relationship showed between STG in red deer and fallow deer density may be mediated by an increased susceptibility and/or exposure at high densities of ungulates due to the competition for scarce resources, but the exact mechanism deserves further research.

### 4.5. Stochastic Factors

Temperature was a significant factor for red deer, displaying the lower STG during the following seasons to the warmest ones. Furthermore, rainfall significantly interacted with temperature to explain STG in fallow deer, so that the effect of the previous season´s temperature on STG was more marked when the previous season was dry. Previous driest and warmest seasons markedly associated with a lower STG (see Figure 6a). Drought together with warm temperatures leads to higher rates of evaporation and the subsequent desiccation, limiting the survival and sporulation of the oocysts in the environment. As consequence, the exposure of herbivores to infective *T. gondii* oocyst decreases [34,35,52].

Additionally, the interaction between rainfall and individual factors was significantly associated with the STG in red deer and wild boar. Concerning red deer, higher previous annual rainfall was related to higher STG in stags, but not in females. This effect of rainy years on the prevalence of pathogens exhibited by males with respect to females have been shown in previous studies on TB in DNP, and may relate to increased exposure and/or susceptibility mediated by sexual behavior and life history traits [14]. An immunosuppressant effect of the intense rut that typically occurs in rainy years has been suggested. Intense rut implies greatest investments by red deer stags in terms of reproductive effort (testosterone metabolite levels and sexual signals), and the conflict between the immune response and the reproductive effort this species is well known [79,80]. *T. gondii* tissue cysts in many organs, including viscera, are believed to persist for the lifetime of the host. In addition, deer rutting typically occurs in the ecotone, which provides excellent wet conditions for oocysts to persist. Despite the same risk derived from the reproductive efforts during the rut exist for fallow deer, no sex-dependent effect of rainfall was observed in STG in this species, which is not surprising since rutting in fallow deer takes place later in Autumn, when rainfall conditions normally are less determinant.

As for wild boar, the positive effect of the rainfall on STG was more markedly in juveniles than adults and piglets. The early dispersal behavior of young males from the natal area may leads to higher exposure to *T. gondii* [81]. This, together with the increased survival of oocysts would give rise to higher infection rates in this age group [35,52].

## 5. Conclusions

This study provides evidence that factors behind the risk of *T. gondii* infection in wild boar, red deer and fallow deer are related to environmental and trophic transmission routes, so as to individual, population and species characteristics. We provided evidence for most of these relationships (e.g., climate or population mediated) and trends. Concomitant pattern among species, indicated that overall, drivers of risk also operated at the community level. However, this research raised several questions that deserve further research. Approximately one-third of the human world’s population is chronically infected while seroprevalence tends to decrease since the early 1960s in many countries [82]. As this decline in seroprevalence leads to loss of immunity, it becomes more relevant for the identification of the epidemiological role of wild host and the understanding of the epidemiology and ecology of *T. gondii* infection in wild host communities and at their interfaces with livestock and human. Thus, game meat, in particular venison, consumption should not be neglected as a public health risk for humans, with the subsequent impact to the public health [31]. For these purposes, addressing host population, community and environmental factors at broad temporal scale is key.

## Figures and Tables

**Table 1:** Results of the GzLMMs of risk factors associated with seroprevalence of *Toxoplasma gondii* in each species studied related to sex, age class, straight-line distance to the nearest coastline (DCOAST), previous season´s rainfall and temperature, annual density of wild ungulates and livestock (horses and cattle), abundance of carnivores and Iberian lynx (KAI), presence of generalized tuberculosis-like lesions (TBL), cover level of closed habitat, straight-line distance to the nearest small human settlements (DHS), and two-ways interactions among them. The model was fitted using sampling season and sampling area as random factors. Parameter estimates for the level of fixed factors were calculated using a reference value of 0 for the male level in the variable sex, calves, and piglets for the variable age in deer species and wild boar, respectively, and negative for the variable presence of generalized TBL.

Variables	Red Deer			Fallow Deer			Wild Boar		
	*F* df (x,y)	Estimate ± SD	*p*	*F* df (x,y)	Estimate ± SD	*p*	*F* df (x,y)	Estimate ± SD	*p*
**Sex ^1^**	0.44 (1, 420)	Female: −3.01 ± 0.99	<0.01				0.22 (1, 695)	Female: 0.89 ± 0.38	0.03
**Age ^2^**	1.46 (2, 420)	Juveniles: 0.01 ± 0.81 Adults: 1.03 ± 0.70	0.04				2.86 (2, 695)	Juveniles: −3.76 ± 1.24 Adults: 0.85 ± 0.76	<0.01
**Presence of generalized TBL ^3^**	2.95 (1, 420)	Positive: 0.59 ± 0.31	0.05	14.47 (1, 446)	Positive: 1.86 ± 0.48	<0.01			
**DCOAST**	25.19 (1, 420)	0.0002 ± 0.00002	<0.01	5.69 (1, 446)	0.00007 ± 0.00003	0.01	26.70 (1, 695)	0.0001 ± 0.00002	<0.01
**DE**	17.67 (1, 420)	0.0002 ± 0.00009	0.07						
**DHS**							2.93 (1, 695)	−0.0001 ± 0.00005	<0.01
**Closed habitat**				5.75 (1, 446)	−0.31 ± 0.15	0.04			
**Previous season´s rainfall**	3.24 (1, 420)	0.01 ± 0.001	<0.01	0.07 (1, 446)	−0.05 ± 0.01	<0.01	2.40 (1, 695)	−0.0006 ± 0.001	0.15
**Previous season´s temperature**	3.23 (1, 420)	−0.68 ± 0.32	0.04	3.88 (1, 446)	−1.61 ± 0.57	<0.01			
**Red deer density**	0.43 (1, 420)	−0.11 ± 0.04	<0.01				1.27 (1, 695)	−0.05 ± 0.03	0.07
**Fallow deer density**	0.43 (1, 420)	0.11 ± 0.06	0.04	6.03 (1, 446)	−0.42 ± 0.18	0.02	7.07 (1, 695)	−0.16 ± 0.07	0.02
**Wild boar density**							4.1 (1, 695)	0.34 ± 0.16	0.04
**Horse density**	1.14 (1, 420)	0.08 ± 0.05	0.10						
**Iberian lynx abundance**				5.81 (1, 446)	3.56 ± 1.60	0.03	0 (1, 695)	−0.86 ± 0.45	0.06
**Carnivores abundance**	13.88 (1, 420)	0.35 ± 0.09	<0.01						
Sex * Rainfall	10.18 (1, 420)	Rainfall * Female: −0.01 ± 0.002	<0.01						
Sex * Age							2.34 (2, 695)	Female * juveniles: −0.97 ± 0.58 Female * ≥ adults: −1.01 ± 0.45	0.07
Temperature * Rainfall				1.95 (1, 446)	0.003 ± 0.0005	<0.01			
Rainfall * Age ^2^							7.19 (2, 695)	Rainfall * juveniles: 0.01 ± 0.002 Rainfall * adults: 0.0003 ± 0.001	<0.01

^1^ Reference value for sex: male, ^2^ Reference value for age: calves (deer species) or piglets (wild boar), ^3^ Reference value for presence of generalized tuberculosis-like lesions (TBL): negative, “*” represents interactions among explanatory variables.

## Supplementary Materials

The following are available online at https://www.mdpi.com/2076-2615/10/12/2349/s1, Table S1: Sample size and seroprevalence of *Toxoplasma gondii* by species, season and sampling site in wild ungulates; Table S2: Summary of the stepwise model selection procedure, based on the AIC, used to explain the serological status against *Toxoplasma gondii*.
