# Supplementary material for: Long-Term Determinants of the Seroprevalence of Toxoplasma gondii in a Wild Ungulate Community
Source: Animals (Basel). 2020 Dec 9;10(12):2349. doi: 10.3390/ani10122349 (PMC7764155; doi:10.3390/ani10122349)
Supplement: Supplementary file 1 [file animals-10-02349-s001.pdf]

Table 1. Sample size and seroprevalence of *Toxoplasma gondii* by species, season and sampling site in wild ungulates and livestock.

Species	Season	CR		SO		RBD		PU		MA	
		N	Seroprevalence (%)	N	Seroprevalence (%)	N	Seroprevalence (%)	N	Seroprevalence (%)	N	Seroprevalence (%)
Red deer	2005–2006	6	0	20	15						
	2006–2007	23	69.6	6	16.7	7	28.6	2	0	8	12.5
	2007–2008										
	2009–2010										
	2010–2011	16	43.8	7	57.1	-		2	0	5	20
	2011–2012	7	71.4	14	64.3	3	33.3	3	0	5	0
	2012–2013	8	100	5	40	3	33.3	1	100	10	20
	2013–2014	4	75	4	50	1	0	-		-	
	2014–2015	16	18.8	8	50	4	0	-		-	
	2015–2016	15	66.7	15	46.7	17	23.5	15	20	15	13.3
	2016–2017	10	40	15	20	15	6.7	15	6.7	15	13.3
	2017–2018	15	60	15	26.7	15	20	15	0	15	6.7
Fallow deer	2005–2006	5	100	13	76.9	-		-		-	
	2006–2007	31	61.3	20	50	8	25	5	80	13	38.5
	2007–2008										
	2009–2010										
	2010–2011	4	75	9	55.6	3	100	7	42.9	7	42.9
	2011–2012	5	60	12	33.3	3	66.7	5	40	6	50
	2012–2013	10	70	6	83.3	2	0	2	0	10	30
	2013–2014	2	100	5	0	2	100	4	25	3	66.7
	2014–2015	1	0	7	0	5	40	5	20	7	0
	2015–2016	15	13.3	15	6.7	13	7.7	18	16.7	16	6.3
	2016–2017	15	40	15	20	14	0	15	6.7	15	0
	2017–2018	12	16.7	15	6.7	18	5.6	15	6.7	15	0
Wild boar	2005–2006			6	16.7	12	8.3	8	25	25	28
	2006–2007	14	42.9	14	21.4	17	29.4	18	38.9	4	25
	2007–2008			26	53.8	7	42.9	13	30.8	27	25.9
	2009–2010	2	50	4	50	17	23.5			18	5.6

	2010–2011	17	58.8	10	50	20	20	12	8.3	9	33.3
	2011–2012	8	75	19	31.6	19	26.3	11	18.2	11	9.1
	2012–2013	9	33.3	6	0	13	0	7	14.3	19	15.8
	2013–2014	-		-		7	0	18	33.3	-	
	2014–2015	2	50	16	6.3	8	37.5	17	37.5	7	28.6
	2015–2016	15	53.3	12	16.7	10	30	6	16.7	8	12.5
	2016–2017	15	46.7	15	26.7	14	14.3	20	10	15	16.7
	2017–2018	14	57.1	14	64.3	13	23.1	13	23.1	14	21.4
Cattle	2010–2011	-		21	4.8	-		27	7.4	27	3.7
	2011–2012	-		-		-		-		21	19
	2012–2013	-		-		12	16.7	-		20	30
	2015–2016	-		23	8.7	32	18.8	55	16.4	41	4.9
	2016–2017	-		-		2	0	-		-	

Table 2. Summary of the stepwise model selection procedure, based on the AIC, used to explain the serological status against *Toxoplasma gondii*.

Model.	AIC
**[M1] full model for red deer: age + sex + generalized_TBL + DE + DCOAST + DHS + DWAT + closed_ha + water_ha + FD_den + RD_den + WB_den + horse_den + cattle_den + KAI_carn + KAI_lynx + rainfall + temperature + sex*age + age*RD_den + rainfall*RD_den + rainfall*sex + rainfall*age + rainfall*temperature**	448.21
M1-DWAT [M2]	446.22
M2-closed_ha [M3]	444.23
M3-rainfall*age [M4]	442.02
M4-age*RD_den [M5]	441.70
M5-water_ha [M6]	439.72
M6-KAI_lynx [M7]	437.87
M7-rainfall*temperature [M8]	436.06
M8-DHS [M9]	434.24
M9-DURB [M10]	432.48
M10-WB_den [M11]	431.19
M11-cattle_den [M12]	430.34
M12-sex*age [M13]	429.48
M13-rainfall*RD_den [final model]	428.39
**[M1] full model for fallow deer: age + sex + generalized_TBL + DE + DCOAST + DHS + DWAT + closed_ha + water_ha + FD_den + RD_den + WB_den + horse_den + cattle_den + KAI_carn + KAI_lynx + rainfall + temperature + sex*age + age*FD_den + rainfall*FD_den + rainfall*sex + rainfall*age + rainfall*temperature**	451.51
M1-age*FD_den [M2]	447.67
M2- rainfall*age[M3]	445.27
M3-age*DE [M4]	443.27
M4-DHS [M5]	441.27
M5-DWAT [M6]	440.05
M6-cattle_den [M7]	438.15
M7-WB_den [M8]	436.22
M8-RD_dens [M9]	434.64
M9-rainfall*FD_den [M10]	433.24
M10-rainfall*sex [M11]	432.09
M11-horse_den [M12]	430.88
M12-DURB [M13]	429.83
M13-water_ha [M14]	429.05
M12-sex*age [M13]	428.50
M13-sex [M14]	426.77
M14-age [M15]	425.25
M15-KAI_carn [final model]	424.92
**[M1] full model for wild boar: age+sex+GeneralizedTBL+DE+DCOAST+DHS+DWAT+closed_ha+water_ha+FD_den+RD_den+WB_den+horse_den+cattle_den+KAI_carn+KAI_lynx+rainfall+temperature+sex*age+age*WB_den+rainfall*WB_den+ rainfall*sex + rainfall*age+rainfall*temperature**	780.79
M1-cattle_den [M2]	770.04
M2- horse_den[M3]	773.84
M3-sex*WB_den [M4]	771.88
M4-KAI_car [M5]	768.09
M5-DWAT [M6]	766.23
M6-DE [M7]	764.56
M7-rainfall*temperature [M8]	763.29
M8-generalizedTBL [M9]	762.15
M9-DURB [M10]	761.39
M10-closed_ha [final model]	761.27
